# Biomarkers of gastric cancer-related ischemic stroke and its underlying pathogenesis

**DOI:** 10.1097/MD.0000000000010493

**Published:** 2018-04-27

**Authors:** Haiyin Long, Kemin Qin, Jiyun Chen, Yicong Chen, Li Chen, Jinsheng Zeng, Zhijian Liang

**Affiliations:** a Department of Neurology, The First Affiliated Hospital of Guangxi Medical University, Nanning City, Guangxi Province; b Department of Neurology, First Affiliated Hospital of Sun Yat-sen University, Guangzhou, Guangdong, P.R. China.

**Keywords:** biomarkers, gastric cancer, pathogenesis, stroke

## Abstract

This study aimed to investigate the biomarkers and underlying pathogenesis of ischemic stroke in patients with gastric cancer (GC).

Patients with active gastric cancer who had experienced acute ischemic stroke without conventional vascular risk factors (gastric cancer-related stroke [GCS] group) and visited The First Affiliated Hospital of Guangxi Medical University and First Affiliated Hospital of Sun Yat-sen University from January 2003 to December 2016 were retrospectively enrolled. The patients’ clinical features and laboratory findings were compared with those of age-, sex-, and disease progression-matched patients with GC without ischemic stroke (GC group) who had been admitted to the same hospital during the same period (GCS:GC ratio = 1:2).

Among the 9166 patients diagnosed with GC, 70 had experienced a cerebral infarction and were enrolled in this study. Among them, 53 (75.71%) harbored multiple lesions in multiple vascular territories. Notably, patients in the GCS group exhibited significant increases in the D-dimer and cancer antigen 125 (CA125) levels and platelet-to-neutrophil ratio (PNR), compared to their counterparts in the GC group. A multiple logistic regression analysis identified all 3 factors as independent risk factors for cerebral infarction in patients with GC (D-dimer, odds ratio [OR] = 1.006 per 1 ng/mL increase, 95% confidence interval [CI], 1.004–1.009, *P* = .000; CA125, OR = 1.016 per 1 U/mL increase, 95% CI, 1.005–1.027, *P* = .005; PNR, OR = 1.025 per 1 point increase, 95% CI: 1.003–1.048, *P* = .023).

Elevated plasma D-dimer and CA125 levels and an increased PNR might affect the occurrence of GC-related ischemic stroke and could therefore serve as potential biomarkers.

## 1 Introduction

Cerebrovascular diseases and malignant neoplasm account for 2 of the top 3 causes of death.^[1]^ Cerebrovascular disease is a frequent comorbidity in cancer patients, of whom 15% experience thromboembolic events during the clinical course of disease.^[2]^ Furthermore, although age, high blood pressure, diabetes, and hyperlipemia are considered common conventional risk factors (CRFs) for cerebrovascular disease, previous studies have suggested that 20% to 40% of cancer patients develop cerebral infarctions despite lacking these CRFs. Accordingly, cancer may itself be an etiologic factor for ischemic stroke. Given this unexplained elevated risk, the identification of etiologic factors is crucial for the treatment and prevention of stroke in cancer patients.

Cancer may induce cerebral infarcts, also known as cancer-related strokes, via an underlying physiopathologic mechanism.^[3–5]^ These infarcts are characterized by significantly elevated plasma D-dimer levels and the development of multiple lesions in multiple arterial territories in the brain in the absence of an identifiable cause of stroke.^[6,7]^ Several cancer-related etiologies of stroke have been suggested, including direct tumor compression or invasion of the blood vessels,^[8,9]^ tumor-induced hemostatic activation or altered blood viscosity,^[10]^ and nonbacterial thrombotic endocarditis.^[11]^ However, increasing evidence suggests that cancer-associated hypercoagulopathy might be an important but underestimated risk factor for cancer-related stroke,^[10,12]^ and this concept is supported by a recent animal study in which mucins produced by carcinoma were found initiate thrombosis through the adhesion-dependent reciprocal activation of neutrophils (NEs) and platelets (PLTs).^[13]^

D-dimer, a degradation product of cross-linked fibrin proteins, is a sensitive marker of hypercoagulation. This marker was previously found to correlate significantly with disease activity and prognosis in many cases of cancer-related stroke.^[3,4]^ A recent study was the first to distinguished cancer-associated acute multifocal embolic infarction from atrial fibrillation using a D-dimer cut-off level of ≥2.0 μg/mL,^[14]^ while another study investigated the potential of the D-dimer level as a predictor of early neurologic deterioration in patients with active cancer and recurrent thromboembolic stroke.^[15]^ The levels of high-sensitivity C-reactive protein, fibrinogen, and probrain natriuretic peptide have also been identified as potential biomarkers of cancer-related stroke.^[16,17]^ To date, most relevant previous studies have been based on case reports, although newer studies are based on clinical data, risk factor analyses, and imaging findings. Overall, however, few studies have attempted to clarify the underlying pathogenesis and biomarkers associated with stroke in a population with a specific cancer type. As these pathogenic factors and biomarkers may vary according to the type of neoplasm, research tailored to specific cancer cell types might provide more reliable results regarding the factors related to stroke.

Gastric cancer (GC) is among the most common malignant neoplasms worldwide and currently ranks 4th and 2nd among all cancers in terms of incidence and mortality, respectively.^[18]^ Notably, a recent study suggested an association of GC with higher risk of ischemic stroke (adjusted hazard ratio [HR] = 1.61).^[19]^ Therefore, with this study, we aimed to clarify our understanding of the pathogenesis and plasma biomarkers of gastric cancer-related stroke (GCS) through comparisons with a population of age- and sex-matched GC patients without stroke.

## 2 Materials and methods

### 2.1 Study design, subjects, and setting

We conducted a retrospective cohort study of adult patients with GC who were diagnosed with acute ischemic stroke or who presented with stroke as the first manifestation of previously undiagnosed GC at The First Affiliated Hospital of Guangxi Medical University and First Affiliated Hospital of Sun Yat-sen University between January 2003 and December 2016. Patients were identified through an administrative database search for International Classification of Diseases, Tenth Revision, Clinical Modification codes, such as C16 and I63 for gastric cancer and ischemic stroke.

Briefly, the GC diagnoses of all patients were pathologically confirmed. Diagnoses of acute stroke were based on the American Heart Association diagnostic criteria for stroke,^[20]^ which included the sudden onset of slurred speech, hemispheric paralysis and limb numbness, or other focal neurological deficits with no cerebral hemorrhage evident on computed tomography (CT) images and the appearance of hyperintense lesions on T2-weighted magnetic resonance (MR) images and diffusion-weighted images. The etiology of stroke was determined according to the TOAST criteria.^[21]^ All methods were applied in accordance with the The First Affiliated Hospital of Guangxi Medical University Committee's guidelines. Ethical approval for the study was obtained from the ethical committee of each participating hospital.

GCS has an unclear pathogenesis and is thus difficult to identify. Based on the definitions of cancer-related stroke used by previous studies,^[3–5]^ we defined GCS as acute ischemic stroke in patients with active GC. Patients were classified into the GCS group if they met the following recruitment criteria determined by 1 oncologist and 1 neurologist who were blind to this study: a previous diagnosis of GC, active disease status (ie, GC treatment had not yet initiated or failed to meet the criteria for a clinical cure, or a recurrence or metastasis of GC had been confirmed), and acute ischemic stroke without CRFs; or initial presentation with acute ischemic stroke, followed by a diagnosis of GC during antistroke therapy. Patients were excluded if they presented with CRFs, primary or metastatic intracranial malignant tumors or other malignant conditions, or cerebral vascular disease other than cerebral infarction.

Patients in the GC alone group were selected after the case group was determined. These patients had been diagnosed with active GC, did not have acute stroke, and were admitted during the same period as the case patients. The exclusion criteria for GC patients included the following: the presence of CRF; brain metastasis; and other cancers. Two age- and sex-matched GC controls were selected for each GCS patient, and the cases and controls were matched according to the GC progression level to balance the groups during the data-collection phase.

### 2.2 Collection of clinical data

We collected demographic data regarding age and sex, CRFs including hypertension (previously diagnosed and treated or a blood pressure ≥140/90 mm Hg), diabetes (previously diagnosed and treated or a fasting glucose level ≥126 mg/dL), hyperlipidemia (previously diagnosed and treated or a fasting serum cholesterol level ≥200 mg/dL or low-density lipoprotein level ≥140 mg/dL), atrial fibrillation, current smoking habit, and patient and family histories of stroke. We also collected clinical course data, including symptom severity, progression, and recurrence, as well as blood test data from routine blood examinations, blood biochemical assays, and inflammation analyses. Additionally, we reviewed each patient's cancer diagnosis, clinical manifestations, pathological type, stage, treatment status, and levels of tumor markers such as carcinoembryonic antigen (CEA), cancer antigen (CA) 125 (CA125), CA153, and CA199. Acute ischemic stroke data, including clinical manifestations, the severity of focal neurological deficits, and post-stroke functional outcomes, were measured using the National Institutes of Health Stroke Scale (NIHSS). Imaging endpoint data, including electrocardiograph (ECG), cranial CT, magnetic resonance images, and diffusion-weighted image findings, were also collected.

### 2.3 Statistical methods

All statistical analyses were performed using SPSS 20.0 software (IBM, Inc, Armonk, NY). An independent sample *t* test was used to compare continuous variables between groups, while Pearson chi-square or Fisher exact test was used to compare categorical variables. A conditional multivariable binary logistic regression analysis was performed to predict the independent contributions of factors associated with GCS versus GC. A *P* value <0.05 was considered statistically significant. Significant variables that received a *P* value <0.05 in the univariate analyses were considered explanatory variables and entered together into multivariable models.

## 3 Results

A total of 70 GCS patients were ultimately enrolled during the study period, accounting for 0.76% of all GC patients. Of these 70 GCS patients, 51 (72.8%) were male and 19 (27.1%) were female, with a mean age (±standard deviation) of 67.53 ± 12.55 years. The majority of patients had a histologic subtype of adenocarcinoma, half had received surgical treatment, and 36 of 70 (51.4%) had an NIHSS score of 5–15. Additionally, 31 patients (44%) presented with metastasis, and 53 (75.71%) had multiple lesions in multiple vascular territories. As expected, the 2 groups did not differ significantly in terms of age or sex. The demographic characteristics, NIHSS scores, cancer subtypes, and time intervals between the diagnoses of cancer and cerebral infarction are summarized in Table 1.

**Table 1:**
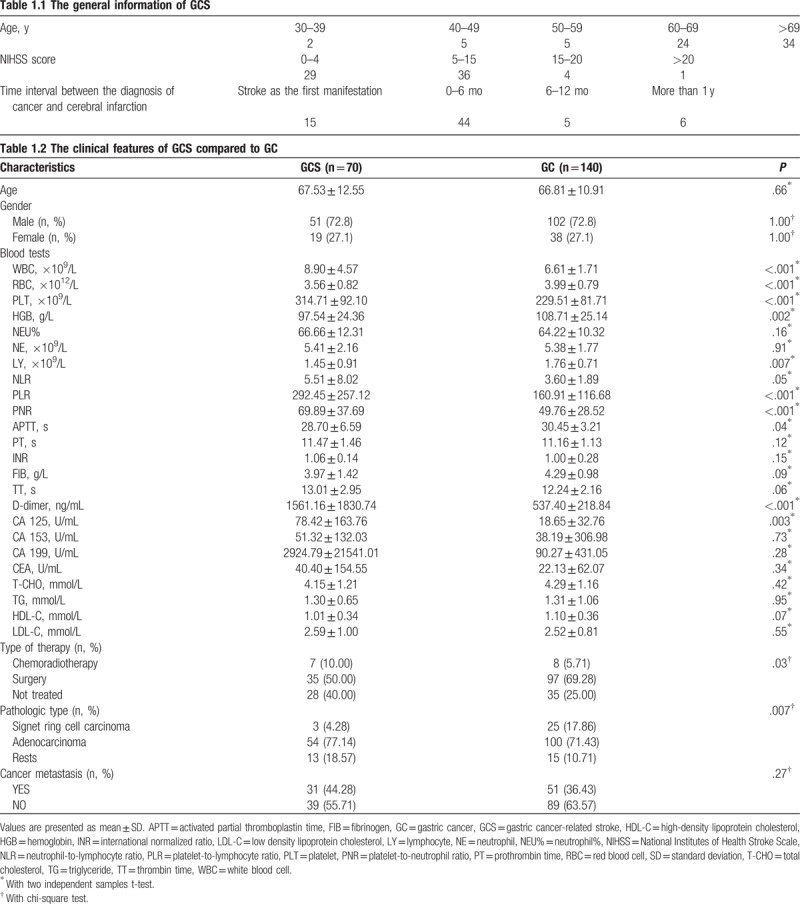
The clinical features of GCS compared to GC.

Of the GCS patients, 15 (21.4%) developed a stroke as the first manifestation of concealed gastric cancer. Furthermore, 44 (62.8%) experienced a stroke during the first 6 months after the GC diagnosis, 5 (7.14%) developed a stroke between 6 months and 1 year after GC diagnosis, and 6 (8.57%) developed stroke >1 year after GC diagnosis. Fifty-three (75.71%) GCS patients presented with multiple lesions on brain magnetic resonance images. Patients with systemic metastasis were similar between the 2 groups. Regarding laboratory findings, however, the 2 groups differed significantly in terms of the white blood cell count, red blood cell count, hemoglobin level, PLT count, lymphocyte count, platelet-to-lymphocyte ratio, platelet-to-neutrophil ratio (PNR), activated partial thromboplastin time, D-dimer, and CA125. Additionally, the groups differed significantly in terms of the type of therapy and pathologic disease type.

To identify independent potential risk factors for stroke in patients with GC, the differential factors mentioned above were subjected to a conditional multivariate logistic regression analysis. Here, the regression coefficients of 3 variables, D-dimer (X1), CA125 (X2), and PNR (X3), were statistically significant and were entered into the final models. The final regression equation was as follows:

logit p = −9.964 + 1.006X1 + 1.016X2 + 1.025X3.Using this equation, the D-dimer level (odds ratio [OR]: 1.006 per 1 ng/mL increase; 95% confidence interval [CI]: 1.004–1.009; *P* = .000), CA125 level (OR: 1.016 per 1 U/mL increase; 95% CI: 1.005–1.027; *P* = .005), and the PNR (OR: 1.025 per 1 point increase; 95% CI: 1.003–1.048; *P* = .023) were identified as independent risk factors for cerebral infarction in patients with GC (Table 2).

**Table 2:**
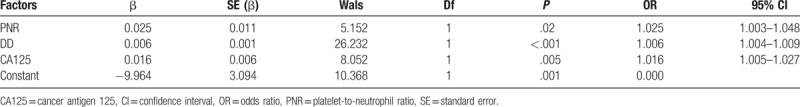
Multivariate logistic regression analysis.

## 4 Discussion

Cancer patients face increased risks of stroke, including recurrent stroke. Zöller et al^[6]^ found that the risk of hospitalization for stroke increased significantly during the first 6 months after a diagnosis of cancer, and identified an overall ischemic stroke risk of 1.6 times higher than that of normal population during this period. In that study, the incidence of cerebral infarction among gastric cancer patients was 1.8%. In our study, GCS was diagnosed in 70 patients, who represented 0.76% of all patients with GC; of these, 59 (84.3%) suffered a stroke within the first 6 months after the diagnosis of GC. The results of our study suggest a general association of cancer, especially newly diagnosed cancer, with an increased risk of subsequent ischemic stroke and suggest that the risk of cancer-related stroke should not be ignored. Further research on cancer-related stroke is admitted of no delay.

To date, the incidence of cancer-related stroke has been shown to associate with cancer-specific mechanisms such as hypercoagulability, nonbacterial thrombotic endocarditis, tumor embolism, infection, and treatment-related mechanisms.^[12]^ However, the pathogenesis of acute cerebral infarction has remained elusive in most cancer patients, as the risk factors and biomarkers of stroke in this population have not been clearly established. A previous study observed fewer vascular risk factors and higher D-dimer levels in cryptogenic stroke patients with active cancer,^[22]^ and the frequency of microembolic signals on transcranial Doppler images correlated linearly with D-dimer levels in this population.^[10]^ In 2016, Ito et al^[14]^ successfully used elevated plasma D-dimer levels to differentiate cancer-related stroke from atrial fibrillation-related acute multifocal embolic stroke, and a recent research identified D-dimer as a predictor of early neurologic deterioration in cryptogenic stroke with active cancer.^[15]^ The association of D-dimer with hypercoagulability suggests that cancer-specific hypercoagulability is the main mechanism underlying cancer-related ischemic stroke. Consistent with this suggestion, the GCS patients in our study had significantly elevated plasma D-dimer levels. Moreover, our multivariate logistic regression analysis revealed that an elevated plasma D-dimer level may independently increase the risk of stroke in GC patients. In brief, the hypercoagulation associated with an increase in D-dimer levels is an important and distinct clinical features of GCS and may also serve as a biomarker of this condition.

As mentioned above, the importance and significance of coagulation disorders in terms of cancer-related stroke remains unclear, and such disorders are difficult to diagnose because common coagulation markers, including D-dimer, lack specificity, and sensitivity. By contrast, CA125 is a mucinous serum marker expressed in patients with various types of cancer, and Elikowski et al^[23]^ demonstrated that in a cancer screening study that a state of hypercoagulation should be considered in patients with a high CA125 level and lung tumors on CT. Similarly, in 2005 Jovin et al^[24]^ reported 4 patients with metastatic cancer, brain infarcts, and markedly elevated CA125 levels and suggested a possible associations between this protein and stroke. Furthermore, animal experiments have determined that mucins secreted by cancer cells trigger the reciprocal activation of PLTs and NEs, leading to the formation of microthromboembolism.^[13]^ Consistent with earlier findings, our study demonstrated that GCS patients had significantly elevated CA125 levels when compared to GC patients, and a multivariate logistic regression analysis revealed that these elevated CA125 levels independently increased the risk of stroke in GC patients. Additionally, 23 patients in our study had elevated levels of both CA125 and D-dimer. Taken together, our and others’ results suggest that GC cells secrete mucinous substances, such as CA125, into the bloodstream to induce a hypercoagulable state, which in turn leads to embolic stroke. Further exploration may verify CA125 as a useful biomarker of GCS.

Although hypercoagulability is common among cancer patients, consensus has not been reached regarding the importance of a hypercoagulable state versus CRFs in the etiology of cerebrovascular disease. Our study observed significant increases in the PLT and PNR in GCS patients relative to GC patients. Moreover, our multivariate logistic regression analysis identified an elevated PNR as an independent risk factor stroke in GC patients. PLTs are multifunctional cells that participate in coagulation, and an increased PLT suggests a hypercoagulable state and a tendency to develop thrombosis. Recently, neutrophil extracellular traps (NETs), which are generated from activated NEs and comprise cell-free DNA, histones, and granular and cytoplasmic NE proteins, have been considered as a novel coagulation-promoting mechanism. The reported rates of secondary thrombocytosis associated with malignant tumors are as high as 30% to 60%,^[25]^ and Yang et l^[26]^ suggests that GC creates a systemic environment that primes NEs to release procoagulant NETs. Therefore, targeting NETs might improve coagulopathy in patients with GC. Animal experiments^[13]^ have determined that mucins secreted by cancer cells trigger the reciprocal activation of PLTs and NEs. Our and previous findings therefore suggest that in GC patients, cerebral infarction may develop as follows: GC cells produce significantly greater amounts of mucins, which inducePLT–NE interactions that lead PLT activation. These earlier steps lead to the formation of a microthromboembolism and, eventually, the occurrence of a cerebral infarction. In other words, the hypercoagulable state induced by activated PLTs plays an important role in GCS.

In summary, our study demonstrated that elevated plasma D-dimer and CA125 levels and an increased PNR induce a hypercoagulable state, which leads to GCS. Notably, these parameters may also be biomarkers of GCS. However, we must also consider the scenario of occult GC, wherein patients without CRFs present with stroke as the first manifestation of malignant disease.

This study had a few limitations of note. First, the study group comprised patients admitted to only 2 hospitals, which may have led to a selection bias. Second, the clinical significance of PNR is not obvious, and the mechanisms underlying cancer-related hypercoagulability are complex, highly variable among individuals, and not fully understood. Furthermore, only 70 patients were ultimately enrolled in this study. We believe that our results warrant a prospective population-based cohort study, which would better address the pathogenesis and biomarkers of cerebral infarction in GC patients. Such a study would allow us to devise various therapeutic treatments that would appropriately manage this condition.

## 5 Conclusions

In summary, elevated plasma levels of D-dimer and CA125 and an increased PNR appear to induce a hypercoagulable state, which may promote the occurrence of GRS. These identified risk factors may also be useful biomarkers of GCS.

## Author contributions

**Conceptualization:** Haiyin Long, Yicong Chen, Li Chen, Zhijian Liang.

**Data curation:** Haiyin Long, Kemin Qin, Jiyun Chen, Yicong Chen, Zhijian Liang.

**Formal analysis:** Haiyin Long, Kemin Qin, Jiyun Chen, Li Chen, Jinsheng Zeng.

**Funding acquisition:** Zhijian Liang.

**Investigation:** Haiyin Long, Kemin Qin, Jiyun Chen.

**Methodology:** Haiyin Long, Kemin Qin, Jiyun Chen, Li Chen, Jinsheng Zeng.

**Project administration:** Zhijian Liang.

**Resources:** Haiyin Long, Yicong Chen, Jinsheng Zeng.

**Software:** Haiyin Long, Li Chen, Zhijian Liang.

**Supervision:** Li Chen.

**Validation:** Jinsheng Zeng.

**Visualization:** Zhijian Liang.

**Writing – original draft:** Haiyin Long.

**Writing – review & editing:** Haiyin Long, Yicong Chen, Li Chen, Jinsheng Zeng, Zhijian Liang.
